# Endoscopic submucosal dissection for early gastric cancer in elderly patients: a meta-analysis

**DOI:** 10.1186/s12957-015-0705-4

**Published:** 2015-10-06

**Authors:** Jin-ping Lin, Ya-ping Zhang, Meng Xue, Shu-jie Chen, Jian-min Si

**Affiliations:** Department of Gastroenterology, Sir Run Run Shaw Hospital, School of Medicine, Zhejiang University, 3 East Qingchun Road, Hangzhou, 310016 Zhejiang People’s Republic of China; Institute of Gastroenterology, Zhejiang University, 3 East Qingchun Road, Hangzhou, 310016 Zhejiang People’s Republic of China; Jiangsu Province Key Laboratory of Anesthesiology, Xuzhou Medical College, Xuzhou, People’s Republic of China; Jiangsu Province Key Laboratory of Anesthesia and Analgesia Application Technology, Xuzhou, Peoples’ Republic of China

**Keywords:** Endoscopic submucosal dissection, Early gastric cancer, Elderly patients, Meta-analysis

## Abstract

**Background:**

The effectiveness of endoscopic submucosal dissection (ESD) has been increasingly reported. However, studies addressing the safety and application value of ESD in elderly patients with early gastric cancer (EGC) were still lacking. This meta-analysis was intended to evaluate the feasibility and safety of ESD in elderly patients with EGC.

**Methods:**

A systematic search was conducted in PubMed, EBSCO, Cochrane Library, EMBASE, and Web of Science. Studies were screened out if data of elderly and non-elderly gastric cancer patients were reported separately. The qualities of included studies were assessed using Newcastle-Ottawa Quality Assessment Scale. The pooled odd ratios (ORs) with 95 % confidence intervals (CIs) were calculated. Statistical analysis was conducted using the Review Manager 5.2 (Cochrane Collaboration, Oxford, UK).

**Results:**

Nine studies (eight in Japan, one in China), including a total of 30,100 lesions, met the inclusion criteria. The “en bloc” and histological complete resection rates of the elderly and non-elderly groups were similar [OR, 0.98, 95 % CI, 0.56 to 1.71; *P* = 0.93 and OR, 0.79, 95 % CI, 0.58 to 1.07; *P* = 0.13, respectively]. As for procedure-related complications, similar perforation rates [OR, 1.19, 95 % CI, 0.94 to 1.51; *P* = 0.15], and bleeding rates [OR, 1.13, 95 % CI, 0.83 to 1.56); *P* = 0.43] between the elderly and non-elderly groups were observed. Whereas, the elderly patients had a higher procedure-related pneumonia rate compared with non-elderly ones [OR, 2.18, 95 % CI, 1.55 to 3.08; *P* < 0.01].

**Conclusions:**

The ESD procedure appears to be a safe technique in elderly patients with EGC while appropriate approach should be taken to avoid procedure-related pneumonia.

## Background

Gastric cancer remains one of the major life-threatening problems worldwide, especially in some eastern Asian countries [1, 2]. Various therapeutic options are available currently, including endoscopic treatment, laparoscopic gastrectomy and conventional open surgery. Early gastric cancer (EGC), defined as lesions confining to the gastric mucosa or submucosa [3], usually has a low risk of lymphatic metastasis, thus allowing radical resection of lesions without lymphadenectomy. Endoscopic mucosal resection (EMR) was firstly introduced for intramucosal gastric cancer. However, if the lesions are larger than 20 mm or have invaded into the submucosa, EMR may lead to piecemeal resection and subsequent recurrence, which has been replaced by endoscopic submucosal dissection (ESD). ESD has been proven to be an effective therapeutic method for EGC. A high “en bloc” resection rate would be achieved if the indications are properly followed [4–6].

Despite the fact that surgical operation is the mainstay in the management of gastric cancer, elderly patients with comorbidities and poor functional capacities might not be able to endure such aggressive surgical trauma. Previous studies reported that the incidence of postoperative complications was quite frequent among elderly patients with EGC [7]. Furthermore, the integrity of the stomach plays a critical role in maintaining a normal condition of elderly patients. Patients after gastrectomy are at risk of vitamin B12 deficiency and anemia [8, 9]. In the past decade, cases of elderly EGC patients undergoing ESD were strikingly increased. However, studies reporting the treatment of EGC by ESD in elderly patients have been published previously, reliable evidence seems still lacking due to small sample sizes. Thus, we performed this systematic review and meta-analysis to evaluate the feasibility and safety of ESD in elderly patients with EGC by comparing with non-elderly patients.

## Methods

### Search strategy and study selection

A systematic search was conducted in PubMed, EBSCO, Cochrane Library, EMBASE, and Web of Science to identify articles published until May 2015. The following search algorithms “((((elderly[Title/Abstract]) OR old[Title/Abstract]) OR geriatric[Title/Abstract])) AND ((((early gastric cancer) OR early gastric neoplasm)) AND (((endoscopic submucosal dissection) OR ESD) OR endoscopic resection))” were used. Besides, the reference lists are manually viewed to obtain additional relevant articles. Search was restricted to English and Chinese literature.

The inclusion criteria were as follows: peer-reviewed studies reporting comparison of elderly and non-elderly patients underwent ESD, full texts were available. The exclusion criteria were as follows: studies of which the measured outcomes were not clearly presented or difficult to calculate, duplicate studies, case reports, review articles, editorials, and letters.

### Data extraction and quality assessment

The articles identified by our search strategy were screened by two independent reviewers (JPL and YPZ). Disagreements were resolved through discussion with other two researchers (SJC and JMS). Extracted data including author, study period, geographical region, number of lesions, “en bloc” resection rates (no piecemeal removal of the lesion) and histologically complete resection rates (no neoplastic cells in lesion edges), perforation, procedure-related bleeding, and procedure-related pneumonia. Perforation was diagnosed intraoperatively or by the presence of free air on plain radiograph or CT images after ESD. Procedure-related bleeding was defined as clinical evidence of bleeding after ESD. Procedure-related pneumonia was defined as new or progressive lung consolidation with clinical symptoms after ESD. The Newcastle-Ottawa Quality Assessment Scale (NOS) was used as a quality assessment tool. Scale varies from zero to nine stars: studies with a score equal to or higher than six were considered methodologically sound.

### Statistical analysis

Dichotomous variables were analyzed using the odd ratio (OR) with 95 % confidence intervals (CIs). Statistical heterogeneity was evaluated using methods described by Higgins et al. [10]. *I*^2^ values between 0 and 25 % suggest low heterogeneity, values above 25 % suggest moderate heterogeneity, and values above 75 % suggest high heterogeneity. Pooled effect was calculated using Mantel-Haenszel test for fixed-effects models (in case of low heterogeneity) or DerSimonian and Laird test for random-effects models (in case of moderate or high heterogeneity) [11, 12]. The potential publication bias based on the procedure-related complications was assessed by conducting the funnel plots. Data analysis was performed using Review Manager 5.2 (Cochrane Collaboration, Oxford, UK). *P* < 0.05 was considered as statistically significant.

## Results

### Characteristics of included studies

**Fig. 1:**
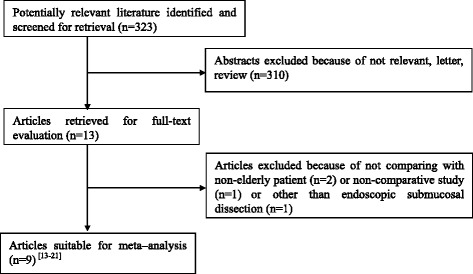
The PRISMA flowchart of literature review

Initially, 323 potentially relevant articles were identified to undergo abstract review. Nine full-text studies were screened out for the final analysis [13–21]. The flow chart of the screening strategies was presented in Fig. 1.

This meta-analysis pooled 30,100 lesions, 6713 in elderly patients group and 23,387 in non-elderly patients group. All of these studies were carried out retrospectively with eight in Japan and one in China. No less than six stars according to the NOS were scored for each of them. The definition of elderly patients was no less than 65 years old in one study [19], no less than 80 years old in another study [20] and no less than 75 years old in the rest seven studies [13–18, 21]. General characteristics, measurements of comparability and the quality of studies were summarized in Table 1.

**Table 1:** Characteristics of included studies

Study	Period	Country	Study design	Group	Sample size	Mean age	Gender (M/F)	Comorbidity (%)	Ulcer findings	Location (U/M/L)	Invasion depth (S/SM)	Tumor size (mm)	Comparability of baseline characteristics	Study quality score
Hirasaki [13]	2000–2004	Japan	R	E	53	78.2	34/19	57	NR	NR	47/6	12.2	abf	6
				NE	91	64.7	74/17	33	NR	NR	83/8	13		
Shimura [14]	2002–2006	Japan	R	E	45	NR	33/8	65.9	NR	6/25/14	NR	16	abc	7
				NE	80	NR	57/18	31.3	NR	11/36/33	NR	NR		
Kakushima [15]	2000–2004	Japan	R	E	49	NR	NR	NR	NR	NR	NR	NR	NR	6
				NE	135	NR	NR	NR	NR	NR	NR	NR		
Onozato [16]	2002–2006	Japan	R	E	110	79.8	50/43	NR	14/96	18/45/47	102/8	22.8	bcef	6
				NE	141	66	106/27	NR	24/117	25/35/81	114/27	21.8		
Isomoto [17]	2001–2007	Japan	R	E	279	NR	173/106	NR	3.7	44/129/105	222/57	20	bcf	6
				NE	434	NR	343/91	NR	13.3	73/209/149	369/65	19		
Toyokawa [18]	2003–2009	Japan	R	E	229	80	128/72	153	11.8	54/76/98	158/28	19	bcdef	8
				NE	357	66	237/77	93.66	10.1	93/141/122	245/41	18		
Tokioka [19]	2002–2010	Japan	R	E	372	73.9	260/112	115.3	NR	25/109/229	367/5	15.1	abcdf	7
				NE	143	57.7	118/25	58.8	NR	23/45/74	138/5	14.5		
Murata [20]	2009–2010	Japan	R	E	5525	NR	3619/1906	61.7	NR	569/2801/2155	NR	NR	NR	6
				NE	21,860	NR	16,657/5203	44.4	NR	1880/12,001/7979	NR	NR		
Zhang [21]	2010–2013	China	R	E	51	79	33/13	76.1	11/51	9/17/24	40/11	19	abcde	8
				NE	136	59.4	79/46	37.6	21/136	9/44/83	129/7	20		

*M* male, *F* female, *L* lower third of stomach, *M* middle third of stomach, *U* upper third of stomach, *E* elderly group, *NE* non-elderly group, *R* retrospective, *NR* not report, *a* gender, *b* tumor size, *c* tumor location, *d* macroscopic types, *e* ulcer findings, *f* invasion depth

### Operative outcomes and procedure-related complications

**Fig. 2:**
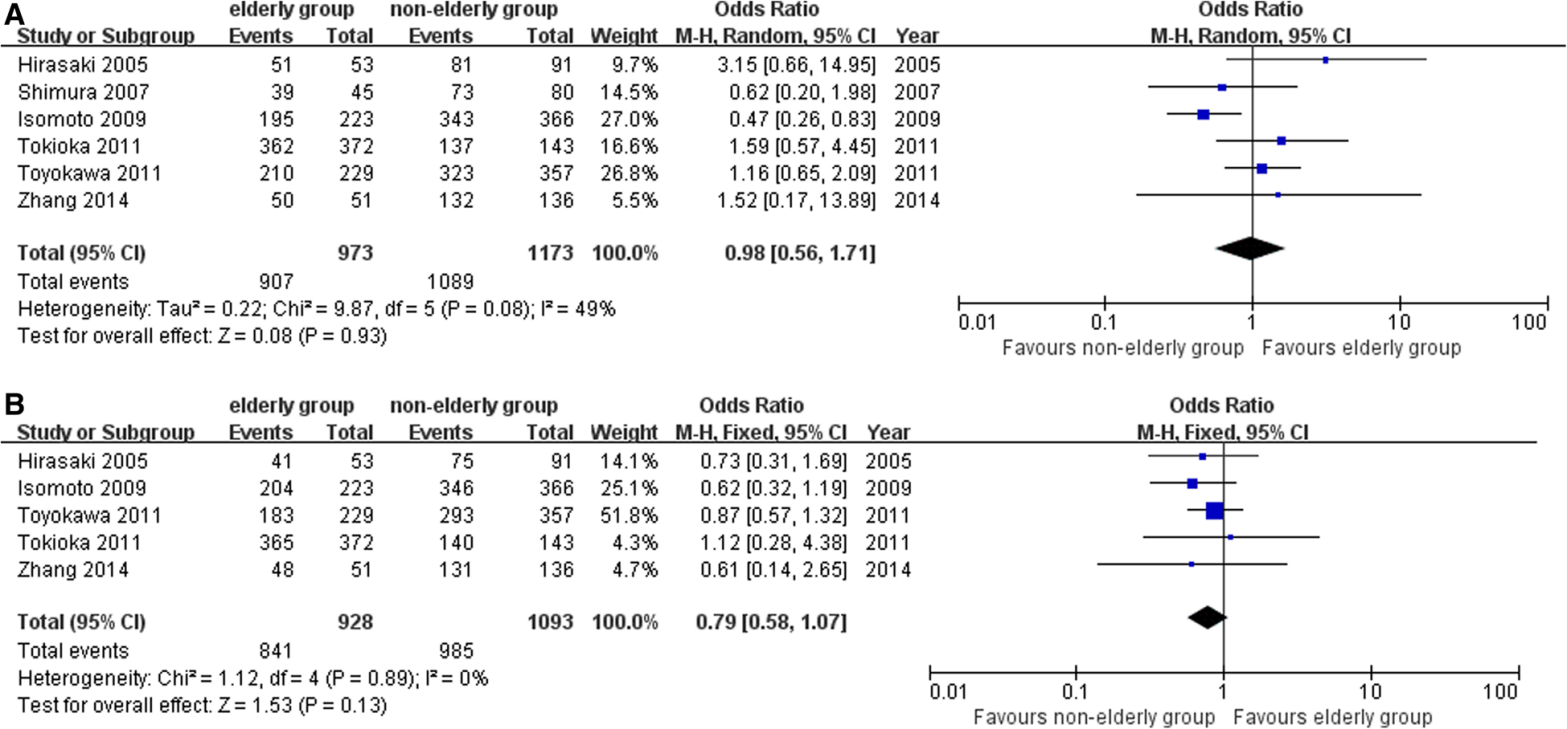
Operative outcomes of the pooled studies (**a** “en bloc” resection rates, **b** histological complete resection rates)

Six studies reported the “en bloc” resection rates [13, 14, 17–19, 21]. In total, “en bloc” resection was performed in 907 out of 973 lesions in the elderly patients group and 1089 out of 1173 lesions in the non-elderly patients group. The “en bloc” resection rates were comparable between the two groups [OR = 0.98; 95 % CI 0.56 to 1.71; *P* = 0.93] (Fig. 2a).

Five studies reported the histological complete resection rates [13, 17–19, 21]. Similar with the “en bloc” resection rates, no significant difference was observed between the two groups [OR = 0.79; 95 % CI 0.58 to 1.07; *P* = 0.13] (Fig. 2b).

**Fig. 3:**
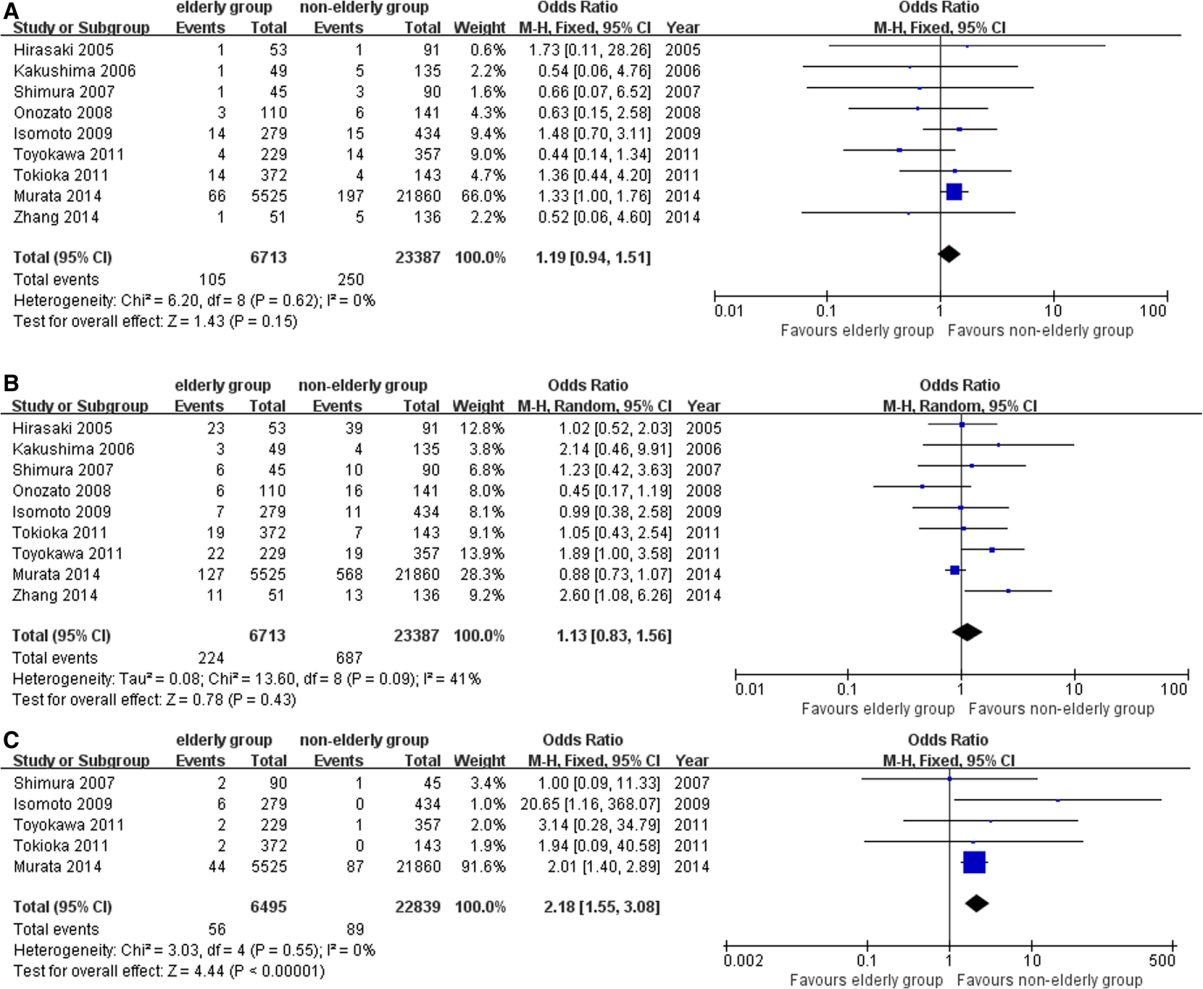
Procedure-related complications (**a** perforation rates, **b** procedure-related bleeding rates, and **c** procedure-related pneumonia)

Data on the perforation rates were reported in nine studies [13–21]. The perforation rate in the elderly patients group (105/6713) was comparable to that in the non-elderly patients group (250/23,387), [OR = 1.19; 95 % CI 0.94 to 1.51; *P* =0.15], (Fig. 3a). Procedure-related bleeding rates were examined in nine studies [13–21] and no significant difference was found between the two groups (elderly vs. non-elderly, 224/6713 vs. 687/23,387), [OR = 1.13; 95 % CI 0.83 to 1.56; *P* = 0.43], (Fig. 3b). Five studies reported the procedure-related pneumonia rates [14, 17–20]. The elderly patients group had a higher risk of procedure-related pneumonia (elderly vs. non-elderly, 56/6495 vs. 89/23,839), [OR = 2.18; 95 % CI 1.55 to 3.08; *P* < 0.01] (Fig. 3c). A summary of operative outcomes and procedure-related complications was showed in Table 2.

**Table 2:** Summary of operative outcomes and procedure-related complications

Study	PT		EBR (%)		HCR (%)		Procedure-related complications							
	E	NE	E	NE	E	NE	E				NE			
							TL	Perforation	Bleeding	Pneumonia	TL	Perforation	Bleeding	Pneumonia
Hirasaki [13]	67	77	96	92	81	82	53	1	23	NR	91	1	39	NR
Shimura [14]	110	NR	81.1	86.7	NR	NR	45	1	2	2	90	3	4	1
Kakushima [15]	NR	NR	NR	NR	NR	NR	49	1	3	0	135	5	4	0
Onozato [16]	NR	NR	NR	NR	NR	NR	110	3	6	NR	141	6	16	NR
Isomoto [17]	NR	NR	93.9	97.9	91.5	94.5	269	7	14	6	434	11	15	0
Toyokawa [18]	123	119	92	90	80	82	229	4	22	2	357	14	19	1
Tokioka [19]	64	77	NR	NR	NR	NR	372	14	19	2	143	4	7	0
Murata [20]	NR	NR	97.3	95.8	98.1	97.9	5525	66	127	44	21,860	197	568	87
Zhang [21]	67.3	37.9	98	97.1	94.1	96.3	51	1	11	NR	136	5	13	NR

*PT* procedure time, *EBR* “en bloc” resection rate, *HCR* histological complete resection rate, *E* elderly group, *NE* non-elderly group, *TL* total lesions, *NR* not report

### Publication bias

**Fig. 4:**
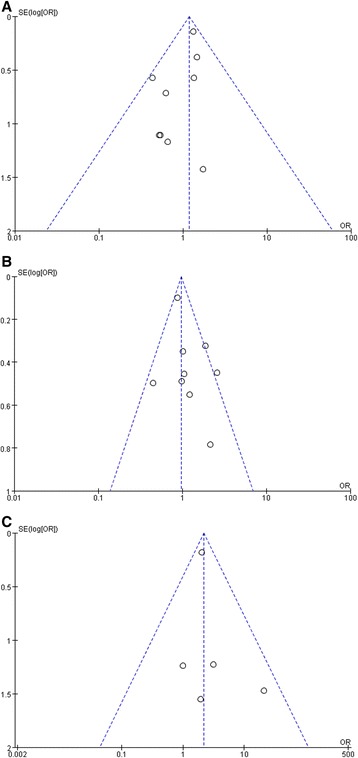
Funnel plots based on the procedure-related complications (**a** perforation, **b** procedure-related bleeding, and **c** procedure-related pneumonia)

The funnel plots based on the procedure-related complications (perforation, procedure-related bleeding and procedure-related pneumonia) were generated (Fig. 4). No evident publication bias was observed. Sensitivity analysis was performed by exclusion of the highest weighted study or the two studies which did not define elderly patients as “no less than 75 years old” [19, 20] in each pooled analysis. The results were all consistent with the outcomes mentioned above.

## Discussion

Owing to the advance in medicine and health care, the global population of the elder has been increasing [22]. An accompanying issue is that neoplastic diseases would be more common, including gastric cancer [23, 24]. Endoscopic treatments have gradually gained their popularity and are currently established as the standard treatment for EGC [25, 26]. ESD is a promising approach of endoscopic treatment, which allows “en bloc” resection for large lesions and recurred less than EMR [27–29]. A meta-analysis pooling ten studies demonstrated the “en bloc” and histological complete resection rates were significantly higher in the ESD compared with EMR [30]. However, ESD is also associated with high frequencies of procedure-related complications, such as perforation, postoperative bleeding, and pneumonia. [29–31]. ESD is expected to be a promising alternative for elderly patients with EGC because of its minimal invasiveness and retainment of integrated stomach when compared with gastrectomy. However, published studies on the application of ESD were not adequately robust to support or refute its feasibility and safety in elderly patients with EGC. Hence, a systematic review pooling the latest evidence was necessary to address this issue.

The long-lasting procedure was one of the drawbacks during ESD. The duration time differed a lot among these studies [13,14, 18, 19, 21]. This can be explained with the learning curve of endoscopists and the locations of tumor which might mostly reside in upper and/or middle portion of the stomach. Factors associated with the longer procedure include locations and sizes of tumor and the presence of ulcer and scar [26, 32]. The influence of age on duration time was assumed to be limited based on the same nature of the procedure, although poor conditions of the elderly patients might need more complex operation. Our present study also showed no significant difference between two groups.

The procedure-related complications are not only preferred parameters to evaluate the feasibility and safety of an operation but also significantly affect the length of hospitalization and medical expenses. Perforation is one of the most common drawbacks accompanying ESD. Perforation after ESD occurs at a rate of 1.2 to 8.2 % [26, 33, 34], even in experienced hands. Our meta-analysis showed that perforation rate was about 2~4 %, irrespective of the patients’ age. Intriguingly, less perforation was reported if the lesions were small and locates at the lower or middle portion of stomach [35, 36]. Nowadays, thanks to the development of endoscopic clipping and prompt use of antibiotics, perforation is no longer an obstacle in most cases.

Procedure-related bleeding was another common complication of ESD procedure. It seemed to be associated with factors including the histology, location, and invasion depth of tumor [13, 37, 38]. For the elder, anticoagulant drugs have long been considered as an important relevant factor. However, recent studies reported that continuous administration of anticoagulant drugs was not significantly correlated with procedure-related bleeding [39, 40]. This meta-analysis revealed the bleeding rates between the elderly and non-elderly groups were similar [OR = 1.13; *P* = 0.43].

According to this meta-analysis, we inferred pneumonia developed more frequently in the elderly patients. Higher risk of aspiration, poor immunity, and less capability to expectorate after ESD contributes to procedure-related pneumonia in the elderly patients. Adequate suction of saliva during ESD might be helpful to reduce the probability of aspiration [16]. Procedure-related pneumonia was also associated with longer operation time, smoking history, sedation methods, and presence of ulceration [41–43]. Thus, elderly patients combined with risks such as smoking, intractable lesions, are recommend to experienced endoscopists, which may avoid procedure-related pneumonia. Moreover, chest radiography images, WBC count, and C-reactive protein level are recommended in elderly patients who are at high risk of procedure-related pneumonia [41, 44]. Though without strong evidence, prophylactic use of antibiotics is recommended in these patients.

Two studies reported the follow-up data after the procedure of ESD in elderly patients, and the long-term prognoses were acceptable. Although only few studies reported long-term oncologic outcomes, the “en bloc” resection rate and the histological complete resection rate are also used as indicators of the oncologic adequacy of ESD [25]. The overall 5-year survival rates in the curative resection and non-curative resection were 85 and 63 % in elderly patients [45]. Both of the “en bloc” resection rate and the histological complete resection rate were high in elderly patients, which were in accordance with the previous reports [26, 46]. Compared with Eastern and Western historical studies, these two parameters of elderly patients were not inferior [27, 47, 48]. This meta-analysis also demonstrated that the “en bloc” resection rate and the histological complete resection rate in the elderly patients were comparable with the non-elderly patients.

Several limitations exist in this meta-analysis. Firstly, eligible studies were all non-randomized controlled trials. A symmetric distribution of lesion size and location, varied indications for ESD, inconsistent definition of elderly patients, and procedure-related complications decreased the plausibility of the results. Secondly, in this meta-analysis, some pooled studies included patients with gastric adenoma [14, 18]. A larger sample size in a meta-analysis may help to obtain a possible treatment effect. The sample size of the rest studies is too small to generalize definitive conclusions of some comparisons. Thus we did not delete these studies, which might be one source of heterogeneity. Thirdly, only studies published in English were pooled in this meta-analysis which may also result in bias. In addition, all nine studies included in this meta-analysis were from East Asia, which may limit its clinical application in Western countries.

## Conclusions

In conclusion, ESD is an effective and safe procedure for elderly patients with EGC, but attentive care should be carried out to avoid procedure-related pneumonia. More well-designed large scale clinical studies are awaited and further evaluation of the utility of ESD elderly patients with EGC should be conducted to confirm our findings.
